# Supplementary material for: The Effect of Cataract Surgery on the Risk of Dementia: A Nationwide Cohort Study
Source: J Clin Med. 2023 Oct 10;12(20):6441. doi: 10.3390/jcm12206441 (PMC10607014; doi:10.3390/jcm12206441)
Supplement: Supplementary file 1 [file jcm-12-06441-s001.zip › jcm-2636458-supplementary.pdf]

Supplementary Table S1 Hazard ratios and 95% confidence intervals for development of dementia (univariate analysis).

**Variables**	**HR (95% CI)**	***p*-value**
1. Cataract surgery		
No	*ref*	<0.0001
Yes	1.352 (1.322, 1.382)	
2. Demographic Factors		
**Age group**		
< 65years	*ref*	
≥ 65years	8.277 (7.961, 8.605)	<0.0001
**Sex**		
Male	*ref*	
Female	1.654 (1.614, 1.695)	<0.0001
**Income**		
Q1 (low)	0.679 (0.655, 0.703)	<0.0001
Q2 (lower-middle)	0.67 (0.649, 0.691)	
Q3 (higher-middle)	0.773 (0.752, 0.793)	
Q4 (high)	*ref*	
2. Systemic Comorbidities		
**Diabetes mellitus**		
No	*ref*	
Yes	1.325 (1.293, 1.358)	<0.0001
**Hypertension**		
No	*ref*	
Yes	1.596 (1.558, 1.634)	<0.0001
**Dyslipidemia**		
No	*ref*	
Yes	0.983 (0.96, 1.006)	0.1517
**Stroke**		
No	*ref*	
Yes	1.988 (1.932, 2.046)	<0.0001
**Depression**		
No	*ref*	
Yes	1.523 (1.483, 1.564)	<0.0001
**Chronic heart disease**		
No	*ref*	
Yes	1.596 (1.558, 1.634)	<0.0001
**Glaucoma**		
No	*ref*	
Yes	0.946 (0.878, 1.019)	0.1443

**Diabetic retinopathy**		
No	*ref*	
Yes	1.139 (1.102, 1.177)	<0.0001
**AMD**		
No	*ref*	
Yes	1.238 (1.197, 1.28)	<0.0001
3 Behavioral Factors		
**BMI Group**		
underweight	1.767 (1.646, 1.896)	<0.0001
normal	*ref*	
overweight	0.821 (0.791, 0.853)	<0.0001
Obese I	0.763 (0.737, 0.791)	<0.0001
Obese II	0.747 (0.684, 0.817)	<0.0001
**Smoking**		
Never smoker	*ref*	
Ex-smoker	0.723 (0.685, 0.763)	<0.0001
Current smoker	0.789 (0.752, 0.828)	<0.0001
**Heavy drink**		
No	*ref*	
Yes	0.538 (0.503, 0.576)	<0.0001
4. Ophthalmic Factors (VA, better eye)		
Good (≥20/66)	*ref*	
Bad (<20/66)	2.263 (2.140, 2.393)	<0.0001

CI: Confidence interval; ref: reference.

* Univariate Cox proportional hazards regression analysis was used to calculate the crude hazard ratios (HRs) and 95% confidence intervals (CIs) for dementia.
